# The Effects of Exercise Training on Glucose Homeostasis and Muscle Metabolism in Type 1 Diabetic Female Mice

**DOI:** 10.3390/metabo12100948

**Published:** 2022-10-05

**Authors:** Caitlin C. O’Neill, Erica J. Locke, Darren A. Sipf, Jack H. Thompson, Erin K. Drebushenko, Nathan S. Berger, Brooke S. Segich, Stephen C. Kolwicz

**Affiliations:** Heart and Muscle Metabolism Laboratory, Health and Exercise Physiology Department, Ursinus College, Collegeville, PA 19426, USA

**Keywords:** glucose metabolism, streptozotocin, insulin, chronic exercise, hyperlipidemia, ketosis

## Abstract

Although exercise training is an important recommendation for the management of type 1 diabetes (T1D), most of the available research studies predominantly focus on male subjects. Given the importance of sex as a biological variable, additional studies are required to improve the knowledge gap regarding sex differences in T1D research. Therefore, the purpose of this study was to examine the role of exercise training in mediating changes in glucose homeostasis and skeletal muscle metabolism in T1D female mice. Female mice were injected with streptozotocin (STZ) to induce T1D. Two weeks after STZ injection, control (CON) and STZ mice were exercise trained on a treadmill for 4 weeks. Aerobic exercise training failed to improve glucose tolerance, prevent the decrease in body weight and adipose tissue mass, or attenuate muscle atrophy in T1D female mice. However, insulin sensitivity was improved in T1D female mice after exercise training. Aerobic exercise training maintained skeletal muscle triglyceride content but did not prevent depletion of skeletal muscle or liver glycogen in T1D mice. Gene expression analysis suggested that T1D resulted in decreased glucose transport, decreased ketone body oxidation, and increased fatty acid metabolism in the skeletal muscle, which was not altered by exercise training. These data demonstrate that 4 weeks of aerobic exercise training of a moderate intensity is insufficient to counteract the negative effects of T1D in female mice, but does lead to an improvement in insulin sensitivity.

## 1. Introduction

The American Diabetes Association (ADA) estimates that 1.25 million American children and adults have type 1 diabetes (T1D) with an incidence rate of 15.9% and 8.6% in males and females, respectively [1]. Previous research has suggested that sex is an important biological consideration in determining the risk of cardiovascular disease and mortality [2] as well as the management [3,4] of patients with T1D. Recent research suggests that sex differences in mitochondrial function exist in individuals with T1D, including a reduction in oxidative phosphorylation capacity in females and increased complex I sensitivity in males [5,6]. However, the contribution of these findings to the pathogenesis or treatment of T1D is unknown. Since the sex gap in biomedical research significantly favors the male population [7,8], additional research exploring the consequences and potential treatments in females with T1D is necessary.

T1D is an autoimmune disease that results in deterioration of the beta cells of the pancreas, leading to hypoinsulinemia and hyperglycemia. To maintain glucose homeostasis, T1D patients require exogenous insulin to allow glucose to enter cells to participate in metabolic processes that produce energy. Glucose uptake within the heart and skeletal muscle is achieved primarily through two transporters, glucose transporter 1 (GLUT1) and glucose transporter 4 (GLUT4) [9]. In the basal state, glucose uptake into muscle tissue is mediated by GLUT1. However, in the insulin-stimulated state or in response to exercise or muscle contraction, GLUT4 translocation to the plasma membrane is critical for skeletal muscle glucose uptake [10]. Insulin stimulation of glucose uptake via GLUT4 involves the insulin receptor pathway, which is dependent upon activation of insulin receptors and downstream mediators including phosphoinositide 3-kinase (PI3K) and protein kinase B (Akt) [11]. In the case of T1D, activation of this pathway requires exogenous insulin to promote glucose uptake. However, glucose uptake via GLUT4 can also be stimulated by AMP-activated protein kinase (AMPK) [12]. Since exercise is a known activator of AMPK, and thus, stimulates glucose uptake independent of insulin signaling [13], exercise training in T1D may present a strategy to regulate glucose homeostasis.

A subgroup of individuals with T1D may develop insulin resistance [14], a condition that has been termed “double diabetes” [15]. Since T1D females have greater deficits in insulin sensitivity than T1D males [4], blood glucose management may require additional pharmacological intervention. Therefore, alternative strategies may be necessary to appropriately manage blood glucose homeostasis. Exercise has been suggested as an effective treatment strategy to regulate T1D and insulin resistance [16]. Moreover, exercise can improve insulin sensitivity in both normal and insulin-resistant subjects who have a family history of diabetes [17]. Clinical data support the notion that exercise improves glycemic control in diabetic male patients [18]. In animal models, exercise training protocols using voluntary wheel or treadmill running from 4 to 12 weeks have been reported to improve blood glucose homeostasis and counteract the negative effects of T1D in male rodents [19,20,21,22,23,24]. However, whether exercise training has similar effects in females with T1D is not clear from the existing literature. Moreover, as the previous studies in male animals and humans were co-treated with insulin, the effectiveness of exercise training independent of insulin cannot be determined.

Given the reduced emphasis of sex differences in diabetes research [25] and the limited studies in T1D females [26], we designed a study to examine the impact of aerobic exercise training independent of insulin therapy on glucose homeostasis and skeletal muscle metabolism in T1D female mice. Using a streptozotocin (STZ) injection strategy, we created a T1D mouse model and treated the female mice with 4 weeks of exercise training on a motorized treadmill. Since female mice are reported to have greater exercise capacity and adapt more positively to exercise training compared to males [27,28], we expected aerobic exercise training to elicit positive changes in glucose tolerance and muscle metabolism in the T1D female model. Overall, the findings of this study have the potential to enhance the understanding of the pathophysiology in T1D females and evaluate the effectiveness of aerobic exercise training in T1D female mice.

## 2. Results

### 2.1. Streptozotocin Dose of 75 mg/kg Induces T1D in Female Mice

**Figure 1:**
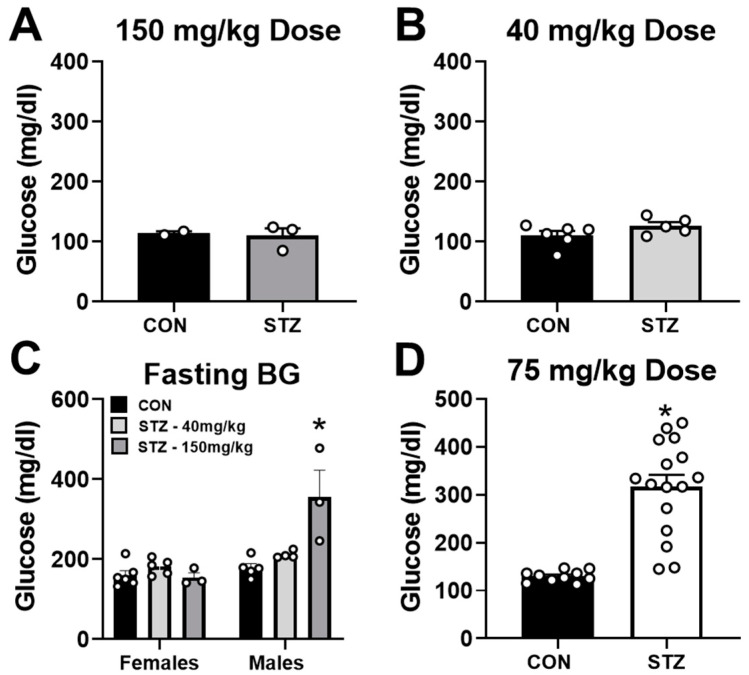
Streptozotocin dose of 75 mg/kg successfully induces T1D in female mice. Three streptozotocin (STZ) injection strategies were attempted in female mice. (**A**) Blood glucose levels in control (CON, n = 2) and STZ (n = 3) female mice 2 weeks after receiving a single injection of 150 mg/kg STZ. (**B**) Blood glucose levels in CON (n = 6) and STZ (n = 5) female mice 2 weeks after receiving a daily injection of 40 mg/kg STZ for 5 days. (**C**) Blood glucose levels in CON and STZ male and female mice 6 weeks after receiving a single injection of 150 mg/kg STZ or a daily injection of 40 mg/kg STZ for 5 days. Females: (CON, n = 6; STZ-40 mg/kg, n = 5; STZ-150 mg/kg, n = 3). Males: (CON, n = 5; STZ-40 mg/kg, n = 4; STZ-150 mg/kg, n = 3). (**D**) Blood glucose levels in CON (n = 10) and STZ (n = 16) female mice 2 weeks after receiving a daily injection of 75 mg/kg STZ for 5 days. * *p* < 0.05 vs. CON.

In pilot experiments, we first elected to inject a single dose of 150 mg/kg BW into a small cohort of female mice. As shown in Figure 1A, this single-high-dose strategy did not lead to a significant increase in blood glucose levels measured two weeks after the injection. We next injected 40 mg/kg BW for 5 consecutive days in a separate small cohort. This strategy also did not cause hyperglycemia two weeks post injections (Figure 1B). To explore whether these two failed injection strategies were a consequence of sex differences, we injected a small cohort of male mice with either a single STZ injection of 150 mg/kg BW or daily STZ injections of 40 mg/kg BW for 5 consecutive days. After 6 weeks, we fasted all mice for 4–5 h and measured blood glucose levels. Surprisingly, only male mice that received a single dose of 150 mg/kg developed significantly elevated fasting blood glucose levels 6 weeks after injection (Figure 1C). Based on a previous publication [29], we attempted a STZ injection strategy using 75 mg/kg BW for 5 days. As shown in Figure 1D, this injection strategy resulted in a significant increase in blood glucose levels >300 mg/dL. These results suggest that the 75 mg/kg multi-dose injection strategy is the most effective for inducing T1D in female mice.

### 2.2. Exercise Training Does Not Improve Glucose Homeostasis in T1D Female Mice

**Figure 2:**
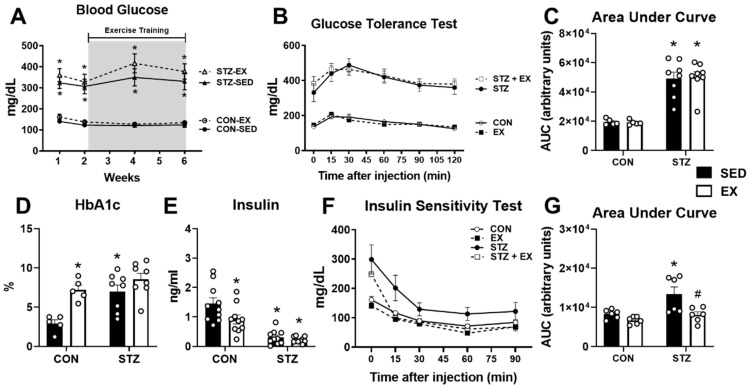
Glucose tolerance and insulin sensitivity in T1D mice after exercise training. Two weeks after completion of the STZ injections, CON and STZ mice were trained on a treadmill for 60 min a day, 5 days/week for 4 weeks. (**A**) Ambient blood glucose levels were monitored from tail tips in female mice. Initially, blood glucose was monitored on a weekly basis in all mice following streptozotocin (STZ) injections (weeks 1 and 2). Thereafter, blood glucose was monitored every two weeks during exercise training (EX). Testing was conducted approximately 16 h after the previous exercise session (n = 5–8 each group). (**B**) Glucose tolerance was tested in a cohort of mice with a glucose solution injection at 1 g/kg of body weight. (**C**) Area under the curve (AUC) analysis of glucose tolerance test curves (n = 5–8 each group). (**D**) Hemoglobin A1c (HbA1c) levels measured in blood obtained from mice after anesthesia and heart removal (n = 5–8 each group). (**E**) Insulin concentrations measured in serum obtained from blood collected from mice after anesthesia and heart removal (n = 10 each group). (**F**) Insulin sensitivity was assessed in a cohort of mice approximately 18 h after the last exercise session. (**G**) Area under the curve (AUC) analysis of insulin sensitivity curves (n = 6 each group). * *p* < 0.05 vs. CON-SED. # *p* vs. STZ-SED.

After confirming hyperglycemia 2 weeks after the completion of the STZ injections, we initiated 4 weeks of exercise training in CON and STZ mice. To assess the effects of exercise training, we monitored ambient blood glucose levels at the midpoint (i.e., 2 weeks) and end of the training (i.e., 4 weeks) and did not observe a significant effect from the exercise training in the CON or STZ groups (Figure 2A). Approximately 18 h after the last exercise training session, a cohort of mice in the sedentary (SED) and exercise training (EX) groups were subjected to a glucose tolerance test (GTT). As shown in Figure 2B, blood glucose levels at baseline were significantly higher in STZ and STZ+EX groups compared to the respective control groups. After injection of glucose, blood glucose was significantly higher throughout the 2 h period in both STZ groups compared to the corresponding control groups (Figure 2B). The area under the curve (AUC) analysis showed no significant differences with exercise training (Figure 2C). HbA1c levels were significantly higher in both STZ groups and were also significantly elevated in the CON+EX group (Figure 2D). As expected, insulin levels were significantly lower in both STZ groups, but were also significantly lower in CON mice after exercise training (Figure 2E). Insulin sensitivity was assessed in a cohort of mice approximately 18 h after the last exercise training session. As shown in Figure 2F and the corresponding AUC analysis in Figure 2G, insulin sensitivity was mildly improved in CON mice and significantly improved in STZ + EX mice. These results suggest that 4 weeks of exercise training of moderate intensity does not improve glucose tolerance but does enhance insulin sensitivity in T1D female mice.

### 2.3. Exercise Training Does Not Protect against the Negative Effects of T1D

**Figure 3:**
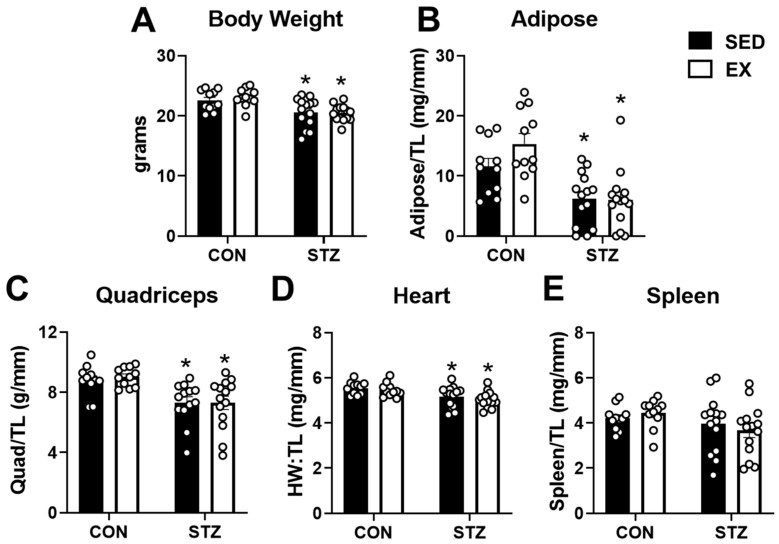
Exercise training does not prevent weight loss and muscle atrophy in T1D mice. (**A**) Body weight; (**B**) mass of inguinal fat pad normalized to tibia length (TL); (**C**) quadriceps mass normalized to TL; (**D**) heart mass normalized to TL (HW:TL); and (**E**) spleen weight normalized to TL obtained from control (CON) and STZ-injected mice after 4 weeks of exercise training (EX). Non-exercised, sedentary (SED) mice were used for comparison. CON, n = 11; EX, n = 11; STZ, n = 14; STZ + EX, n = 14. * *p* < 0.05 vs. CON-SED.

To gain further insight into the effects of T1D and the potential of exercise training to mitigate the response, we evaluated body weight and tissue masses in all groups. As shown in Figure 3A,B, STZ injections led to a significant decrease in body weight and adiposity at the end of 6 weeks. Exercise training in the STZ-treated mice did not prevent body weight and adipose tissue loss (Figure 3A,B). Furthermore, STZ injections in both SED and EX groups decreased quadriceps and heart mass (Figure 3C,D). A reduction in spleen weight has been associated with chronic stress due to exercise training in rodents [30]. As shown in Figure 3E, spleen weight normalized to tibial length was not significantly affected by exercise training or STZ injections. In summary, induction of T1D with STZ leads to significant reductions in body weight and adiposity. In addition, T1D leads to cardiac and skeletal muscle atrophy. All told, these data suggest that 4 weeks of exercise training is insufficient to counteract the negative consequences of T1D in female mice.

### 2.4. Exercise Training Does Not Prevent Dyslipidemia in T1D

**Figure 4:**
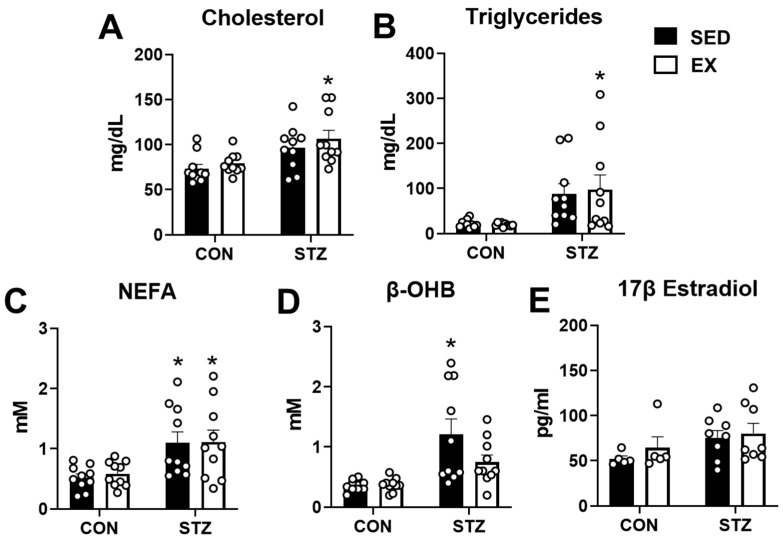
Exercise training does not attenuate hyperlipidemia in type 1 diabetic mice. (**A**) Serum cholesterol; (**B**) serum triglycerides; (**C**) serum non-esterified fatty acids (NEFA); obtained from blood collected from control (CON) and STZ-injected mice after 4 weeks of exercise training (EX) or non-exercised, sedentary (SED) mice (n = 10 each group). (**D**) Beta-hydroxybutyrate (β-OHB) measured in blood obtained from tail tip (n = 10 each group). (**E**) 17-β estradiol levels measured in the serum obtained from blood collected from a cohort of mice after anesthesia and heart removal (n = 5–8 each group). * *p* < 0.05 vs. CON-SED.

To determine the effects of abnormal blood glucose metabolism due to hypoinsulinemia on lipid and ketone body metabolism, we performed measures of serum lipids and beta-hydroxybutyrate (β-OHB) in the mice. Serum cholesterol, triglycerides, and non-esterified fatty acids (NEFA) were all elevated in STZ-treated mice; however, exercise training in CON and STZ groups had no effect on the high lipid levels (Figure 4A–C). β-OHB was also elevated in STZ-treated mice relative to both CON groups (Figure 4D). Interestingly, exercise training in the STZ groups tended to reduce β-OHB concentrations (*p* = 0.11, Figure 4D). Since sex hormones have been associated with alterations in lipid and glucose metabolism [31,32], we measured serum 17β estradiol levels in a cohort of mice and found no statistical difference between the groups (Figure 4E). These results show that exercise training fails to reduce the STZ-induced hyperlipidemia but may reduce ketosis in female mice.

### 2.5. Exercise Training Prevents Depletion of Triacylglycerol Content in T1D Skeletal Muscle

**Figure 5:**
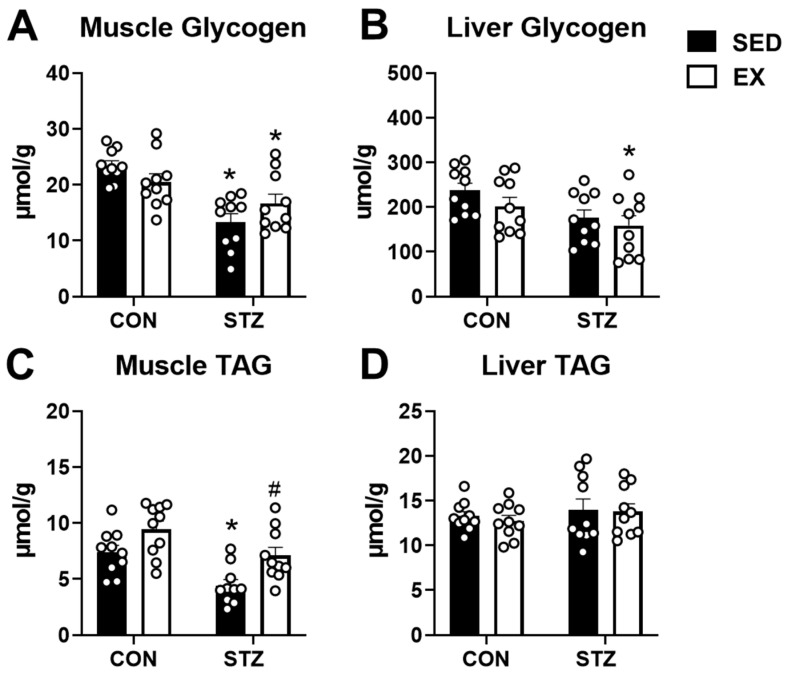
Glycogen and triacylglycerol contents in skeletal muscle and liver of type 1 diabetic mice. (**A**) Glycogen content in quadriceps muscle; (**B**) glycogen content in liver; (**C**) triacylglycerol (TAG) content in quadriceps muscle; and (**D**) TAG content in liver. Tissues harvested from control (CON) and STZ-injected mice after 4 weeks of exercise training (EX) or non-exercised, sedentary (SED) mice (n = 10 each group). * *p* < 0.05 vs. CON-SED. # *p* < 0.05 vs. STZ-SED.

Glycogen and triacylglycerol (TAG) are important endogenous fuel sources within tissues, particularly during exercise. Therefore, we measured both glycogen and TAG within the quadriceps and liver of the mice. STZ injections in both SED and EX mice led to significant reductions in glycogen content in both the quadriceps and liver (Figure 5A,B). Four weeks of moderate exercise training did not significantly alter glycogen content in the skeletal muscle or liver of CON mice (Figure 5A,B). Skeletal muscle TAG content was significantly lower in the STZ-SED group, but was significantly elevated in the STZ + EX group (Figure 5C). TAG content in the liver was not affected by exercise training or STZ (Figure 5D). These data demonstrate that T1D due to STZ injections results in significant depletion of both glycogen and TAG content in the skeletal muscle. Glycogen content of the liver is also depleted in T1D. Although exercise training is incapable of preventing the decreases in skeletal muscle or liver glycogen, depletions in skeletal muscle TAG content may be restored in T1D female mice.

### 2.6. Exercise Training Does Not Prevent Altered Gene Expression in Female T1D Mice

**Figure 6:**
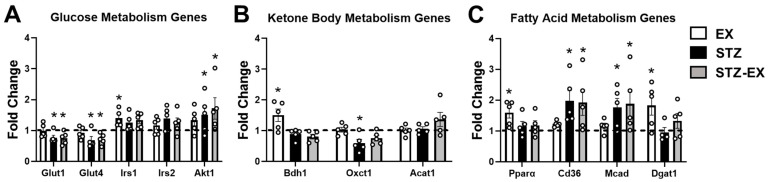
Gene expression in skeletal muscle of type 1 diabetic mice. Expression of genes involved in (**A**) glucose metabolism; (**B**) ketone body metabolism; and (**C**) fatty acid metabolism. mRNA was isolated from gastrocnemius muscles harvested from control (CON) and STZ-injected mice after 4 weeks of exercise training (EX) or non-exercised, sedentary (SED) mice. Glucose metabolism: *Glut1*, glucose transporter 1; *Glut4*, glucose transporter 4; *Irs1*, insulin receptor substrate 1; *Irs2*, insulin receptor substrate 2; *Akt1*, AKT serine/threonine kinase 1. Ketone body metabolism: *Bdh1*, 3-hydroxybutyrate dehydrogenase 1; *Oxct1*, 3-oxoacid CoA-transferase 1; *Acat1*, acetyl-CoA acetyltransferase 1. Fatty acid metabolism: *Pparα*, peroxisome proliferator-activated receptor alpha; *Cd36*, cluster of differentiation 36; *Mcad*, medium-chain acyl-CoA dehydrogenase; *Dgat1*, diacylglycerol acyltransferase 1. Data presented as fold-change over CON-SED group. CON-SED group represented as dotted line. CON, n = 5; EX, n = 5; STZ, n = 5; STZ + EX, n = 5. * *p* < 0.05 vs. CON-SED.

To determine whether exercise training influenced metabolic pathways in T1D female mice, we measured the expression of genes involved in glucose, ketone body, and fatty acid metabolism in gastrocnemius muscle. Glucose transporter 1 (*Glut1*) and glucose transporter 4 (*Glut4*) were downregulated in both STZ and STZ + EX (Figure 6A). Exercise training caused upregulation of insulin receptor substrate 1 (*Irs1*) in EX and STZ + EX (main effects of exercise, *p* < 0.05), whereas AKT serine/threonine kinase 1 (*Akt1*) was upregulated in both STZ groups (Figure 5A). Although 3-hydroxybutyrate dehydrogenase 1 (*Bdh1*) was upregulated in the EX-group, both Bdh1 and 3-oxoacid CoA-transferase 1 (*Oxct1*) were downregulated in the STZ and STZ + EX groups (main effects of STZ injections, *p* < 0.05, Figure 5B). Expression of peroxisome proliferator-activated receptor alpha (*Pparα*) and diacylglycerol acyltransferase 1 (*Dgat1*) were significantly increased in the EX-group, whereas cluster of differentiation 36 (*Cd36*) and medium-chain acyl-CoA dehydrogenase (*Mcad*) were significantly elevated in both STZ and STZ + EX groups (Figure 5C). These findings suggest that STZ injections result in aberrant glucose, ketone body, and fatty acid metabolism in the skeletal muscle of female mice, which is not prevented by exercise training.

## 3. Discussion

There are several important findings obtained from the experiments conducted in the present study. First, this study indicates that a STZ injection of 75 mg/kg per body weight for 5 days is an effective strategy to induce T1D in female mice. Second, hypoinsulinemia resulting in chronic uncontrolled hyperglycemia leads to numerous negative side effects in female mice including weight loss, extreme fat loss, muscle atrophy, dyslipidemia, ketosis, and altered skeletal muscle gene expression. Last, aerobic exercise training without insulin therapy is insufficient to improve glucose tolerance and counteract the negative sequelae of T1D in female mice. However, exercise training does improve insulin sensitivity in T1D female mice. Overall, these findings highlight the importance of insulin therapy to maintain blood glucose homeostasis and suggests that 4 weeks of aerobic exercise training can significantly enhance insulin sensitivity in female mice with T1D.

In general, most studies involving rodents traditionally use male mice for a variety of reasons [8]. In experimental mouse studies investigating T1D, STZ injection strategies utilize either a single-high-dose injection of STZ or a multiple-low-dose injection over the course of 5 consecutive days [33]. In our preliminary studies, neither of those dosing strategies led to significant hyperglycemia in female mice. The failure of these doses is likely due to the protective effect of estradiol on beta-cell survival and the prevention of insulin-deficient states [34]. However, T1D has been achieved successfully in females utilizing STZ dosages higher than males [29]. In accordance with this previous report, approximately 80% of the female mice injected with STZ in our study have blood glucose levels greater than 200 mg/dL two weeks after the completion of the final injection.

STZ injections have documented negative effects on multiple organs and body weight loss [35,36,37,38]. Hyperglycemia due to hypoinsulinemia is linked to a decline in skeletal muscle mass in both human and animal models. Although the exact mechanistic link is unknown, the insulin signaling pathway has been identified as an important contributor to cardiac and skeletal muscle growth via the Akt/mTOR pathway [39,40]. In the current study, we did observe that uncontrolled hyperglycemia induced by STZ leads to an approximate 10% reduction in cardiac mass and a 20% reduction in quadriceps mass in the female mice. Interestingly, we observed a 1.5- to 1.7-fold increase in Akt1 gene expression in the gastrocnemius of both sedentary and exercise-trained mice treated with STZ. Perhaps, this is a compensatory response to counteract skeletal muscle atrophy. In addition to the loss of muscle mass, the body weight was approximately 10% lower with an approximate 50–60% reduction in adipose tissue mass after 6 weeks. In STZ-treated male mice, weight loss of up to 40% after 2 weeks has been reported [41]. Although studies in rats suggest that females may be protected against body weight loss after STZ injections [42,43], our findings in mice are not consistent with this observation. Future studies investigating the potential sex differences in response to uncontrolled T1D could provide some additional insight.

In addition to changes in body weight, STZ-injected mice experienced ketosis and elevated levels of cholesterol, triglycerides, and fatty acids. This finding is not surprising given the large reduction in adipose tissue. In an insulin-deficient state, cellular glucose metabolism is impaired, and therefore, alternate sources of fuel, such as ketone bodies and lipids, are required. During lipolysis, triglycerides are released from the adipose tissue, which likely accounts for the increased concentration of fatty acids and triglycerides in the serum. In addition, the elevated serum fatty acid levels would contribute to increased serum ketone bodies due to ketogenesis in the liver [44]. Excess fatty acids and triglycerides may also be responsible for the high serum cholesterol levels through elevated synthesis in the liver [45]. Although exercise training has been noted to elicit many positive benefits in type 2 diabetes, including a modest improvement in blood lipid levels [46], dyslipidemia in T1D has been attributed more to poor glycemic control [45,47]. Since exercise training in STZ mice has little effect on blood glucose homeostasis, the lack of improvement in the blood lipid profile is not unexpected.

Cellular glucose uptake is achieved through glucose transporters on the cell membrane in heart and skeletal muscle. The two prominent glucose transporters are glucose transporter 1 (GLUT1) and glucose transporter 4 (GLUT4) [9]. GLUT1 is always present on the cell membrane and responsible for most of the basal glucose uptake and functions independent of insulin [48]. On the other hand, the presence of GLUT4 on the cell membrane is influenced by an insulin-activated signaling cascade or exercise [49,50]. Induction of T1D using STZ in male rodents was associated with decreased GLUT4 expression in the skeletal muscle [51], which is consistent with our finding of GLUT4 downregulation in female mice. In our original hypothesis, we predicted that exercise training in the STZ model would improve glucose homeostasis through exercise-induced glucose uptake. In this mechanism, the activation of AMPK via muscle contraction would result in the translocation of GLUT4 to the cell membrane to increase glucose uptake [49,50]. However, 4 weeks of training in our study did not prevent the downregulation of genes involved in glucose transport in the skeletal muscle of female mice treated with STZ. Previous studies in male rodents exposed to voluntary running wheels for 6–8 weeks showed improvements in blood glucose control [23,24]. In addition, exposure to voluntary wheel running for 4 weeks prior to injections of STZ was effective in preventing fasting hyperglycemia in male mice [19]. Since voluntary wheel running is a training modality without a fixed intensity or duration, perhaps a total volume of physical activity represents a more appropriate method to control hyperglycemia. However, a combined resistance and high-intensity aerobic training program of 6, 10, or 12 weeks was effective in improving glucose metabolism and preventing deleterious effects of STZ injections in male rats [20,21,22]. Of note, the above studies also incorporated insulin treatments, which may be critical in mediating the effects of exercise training in this model. Additional studies evaluating the effects of training modality and exercise intensity in T1D females, with and without insulin therapy, are clearly warranted.

Our study of T1D female mice revealed a lack of effect of 4 weeks of aerobic exercise training on glucose homeostasis. In addition to the gene expression analysis of glucose transporters, we performed GTTs within 24 h of the last exercise session as well as biweekly blood glucose tests. Neither of these experiments showed positive changes in glucose homeostasis. Moreover, we measured HbA1c levels, used as a clinical marker of chronic hyperglycemia [52], which was also not affected by aerobic exercise training in T1D females. However, reductions in HbA1c levels are dependent upon the replacement of red blood cells (RBCs). In mice, the RBC lifespan averages ~45 days (half-life is ~22 days) [53], thus it is possible that the length of the study was not sufficient to detect significant changes. Surprisingly, we observed a significant increase in HbA1c levels in the exercise training group. The reason for this observation is not clear. Interestingly, chronic stress has been associated with hyperglycemia and elevated HbA1c levels in mice [54], which could suggest an issue with the exercise training protocol. However, our selected frequency of training (i.e., 5 days per week), duration of training (i.e., 60 min per day), and length of training (i.e., 4 weeks) are all consistent with previously published protocols [55,56,57]. We based the intensity on the reported critical running speed, which is estimated to equal approximately 75% of maximum oxygen consumption (VO_2_ max) [58], and titrated the running speed to an approximate 60–65% intensity, which is consistent with the previous literature [55,57]. Furthermore, the control exercise training group did not experience a significant reduction in spleen weight, which has been suggested to serve as a marker of chronic stress during exercise training [30,59]. Therefore, it is unlikely that the exercise training protocol represented a condition of chronic stress.

In addition to the examination of glucose homeostasis with exercise training in T1D mice, we evaluated other aspects of skeletal muscle metabolism including endogenous storage and the expression of genes involved in glucose, lipid, and ketone body metabolism. Our findings demonstrate that two important endogenous sources of glucose and fatty acids in the skeletal muscle (i.e., glycogen and triacylglycerol, respectively) are significantly reduced in T1D female mice. The phenotype of reduced skeletal muscle triglyceride content is normalized by 4 weeks of exercise training. Our findings also reveal that the mRNA expression of genes involved in ketone body oxidation (i.e., *Bdh1* and *Oxct1*) is reduced in T1D males. Downregulation of the ketone body metabolism pathway from STZ-injected male rodents has been reported in skeletal muscle [60] and heart [61]. Besides a potential reduction in skeletal muscle ketone body oxidation, female mice injected with STZ experience an upregulation of genes involved in fatty acid uptake and oxidation. These findings of increased lipid metabolism are also consistent with previous reports in male rodents [60,62]. Of note, *Bdh1*, *Pparα*, and *Dgat1* were upregulated in the CON-EX group. Since upregulation of these genes have been reported in the skeletal muscle after chronic aerobic exercise training [62,63], the intensity and duration of the exercise training protocol was likely sufficient to induce physiological adaptations, at least in healthy animals. In total, T1D in female mice appears to result in an increased reliance on both exogenous and endogenous fatty acid oxidation at the expense of glucose and ketone body metabolism, which is not reversed by 4 weeks of moderate aerobic exercise training.

The development of insulin resistance in T1D has become a more notable feature in the pathology of T1D and may be associated with excess weight gain, genetic and physiological factors, and treatment-related issues [64]. Evaluation of insulin resistance can be challenging as changes in insulin sensitivity may be related to tissue- or organ-specific differences [65]. Analysis of glucose tolerance and insulin sensitivity did not yield significant differences in the CON-EX group, although it should be noted that changes in glucose tolerance from exercise training could be more related to the acute effects of exercise rather than the chronic effect of training [66,67]. However, fasting serum insulin was lower and the insulin receptor (*Irs1*) was upregulated in the CON-EX group, which suggest that exercise training resulted in some aspects of enhanced insulin sensitivity in the control group. More importantly, insulin sensitivity in the STZ group was significantly improved after 4 weeks of exercise training. These findings highlight the significance of incorporating exercise training into the treatment of T1D since the modification of insulin therapy is an important consideration in the management of T1D.

There are several limitations of our study that should be noted. First, although we included male mice in the determination of the appropriate STZ injection strategy, we did not include male mice in the exercise training study. Therefore, we cannot determine whether our findings are due to sex differences. Second, the exercise training protocol used in our study was consistent with previously published protocols [19,55,56,57]; however, whether an increased length of training could have produced different results is unclear. Third, we did not provide any insulin therapy to the T1D mice in the study or include a group with insulin therapy. Since insulin therapy is recommended for the management of T1D [68], there is a possibility exercise training combined with insulin therapy could have elicited alternate findings. Last, our metabolic assessments in this study were limited to serum, skeletal muscle, and liver. Since exercise training in T1D females may exert positive benefits on other tissues and organs [69], other aspects of health affected by hypoinsulinemia/hyperglycemia may have been improved.

In summary, we attempted to counteract the negative side effects of T1D including poor glucose control, dyslipidemia, and tissue atrophy through 4 weeks of aerobic exercise training in female mice treated with STZ. We specifically tested this hypothesis in female mice considering the sex gap in biomedical research [8]. Although induction of T1D using the STZ model has been reported to be difficult in females, we were successful in generating a T1D female model. All mice treated with STZ were able to complete the 4 weeks of aerobic exercise training despite hyperglycemia, muscle atrophy, and altered skeletal muscle metabolism. Although the exercise training protocol was ineffective at preventing the negative side effects of T1D, which could be due to biological sex differences, the intensity and duration of the exercise protocol, or the lack of insulin therapy, insulin sensitivity was maintained in T1D female mice. Since exercise training is a critical strategy in the management of T1D, additional studies are required to better understand the effects of aerobic exercise training in T1D female mice.

## 4. Materials and Methods

### 4.1. Animal Model

All experimental protocols used in this study were approved by the Institutional Animal Care and Use Committee (IACUC) of Ursinus College (Animal Welfare Assurance: #D16-00678; IACUC Protocol: #2017-3ModA-SK). Fifty female C57BL/6 mice were purchased from a commercial vender (Charles River Laboratory, Stone Ridge, NY) at 10 weeks of age. The mice were randomly assigned to either the control (CON) group or the group injected with streptozotocin (STZ). After injection, CON and STZ mice were randomly assigned to sedentary, non-exercised (SED) or exercise training (EX) groups to yield 4 groups: CON-SED; CON-EX; STZ-SED; STZ-EX. At the start of the study, additional animals were added to both STZ groups to account for any animals that needed to be excluded from the study. No STZ-SED mice were removed due to complications and all STZ-EX mice completed the exercise training protocol. All mice were kept in a 12 h light/dark cycle and received food and water ad libitum.

### 4.2. Streptozotocin Injections

To induce T1D, STZ was injected, which targets and destroys the insulin-producing beta cells of the pancreas. STZ is an antibiotic produced by the bacterium *Streptomyces achromogens*, which contains a glucose molecule that is linked to a highly reactive methyl nitrosourea moiety that is thought to exert STZ’s toxic effects. STZ was purchased from a commercial vendor (Sigma-Aldrich, St. Louis, MO, USA) and prepared in a 50 mM sodium citrate solution at a concentration of 12.5 mg/mL [70]. Mice received an intraperitoneal injection of 75 mg/kg per body weight once a day over the course of 5 days, as previously published [29]. Control mice were injected with a vehicle of sodium citrate. During the injection period and throughout the study, all mice were maintained on normal chow and drinking water. Confirmation of hyperglycemia was made one week after the completion of the injection protocol by assessing the blood glucose obtained from the tail tip as described below.

### 4.3. Blood Glucose Assessment

For the first 2 weeks following the completion of STZ injections, blood glucose was assessed in all mice on a weekly basis via a hand-held glucometer (Contour next EZ, Ascensia Diabetes Care) using blood obtained from the tail tip. Blood glucose was also assessed at 2-week intervals during the exercise training protocol. Blood glucose was measured in the fed state at approximately 1300 h.

### 4.4. Exercise Training Protocol

Two weeks after the completion of injections, mice assigned to the aerobic exercise training groups (CON-EX and STZ-EX) entered an exercise training program for 4 weeks. The aerobic exercise training program consisted of running on a motorized treadmill at moderate intensity for 60 min a day, 5×/week, at 15 m/min with a 10% incline. The treadmill speeds were graded starting at 10 m/min for 10 min, 12 m/min for 10 min, and 15 m/min for 40 min. All exercise sessions occurred between 1600 and 1900 h, near the end of the light cycle (0600 to 1800 h). During the exercise session, animals were motivated to run via a gentle prodding of their hindquarters. Mice in the CON-SED and STZ-SED groups were transported to the animal exercise facility for each session, but remained in their cages during the exercise time.

### 4.5. Glucose Tolerance Test

At the end of 4 weeks of exercise training, glucose tolerance tests were conducted approximately 18 h after the last exercise session. After measuring the baseline blood glucose, the mice received an intraperitoneal injection with a glucose solution of 1 g/kg per body weight. After injection, blood glucose was measured at 15, 30, 60, 90, and 120 min to monitor the blood glucose to near baseline levels. After the glucose tolerance test, mice were returned to the vivarium for a 48 h recovery period.

### 4.6. Insulin Sensitivity Tests

At the end of 4 weeks of exercise training, insulin sensitivity tests were conducted approximately 18 h after the last exercise session. Mice were fasted for 3–4 h prior to initiation of the test. Baseline blood glucose levels were obtained from tail nicks using a hand-held glucometer. Mice were then injected with an insulin solution of 1.0 U/kg per body weight. Blood glucose was measured at 15, 30, 60, and 90 min after the insulin injection. After the insulin sensitivity tests were completed, mice were returned to the vivarium for a 48 h recovery period.

### 4.7. Tissue Harvest

Mice were injected with approximately 170 mg/kg of sodium phenobarbital. After confirming negative on the toe pinch test, the following procedures were conducted. The heart was removed and rinsed in ice cold phosphate-buffered saline (PBS). The heart was trimmed of excess tissue, weighed, and the left ventricle was dissected and frozen in liquid nitrogen. The quadriceps, adipose tissue, and spleen were removed, blotted dry, and weighed. A piece of liver, soleus, and gastrocnemius were removed, blotted dry, and frozen in liquid nitrogen. The tibia was dissected and measured to normalize tissue weights. All tissues were stored at −80 °C until analysis.

### 4.8. Serum Analysis

Beta-hydroxybutyrate (β-OHB) is the most abundant ketone body in the blood [44]. Therefore, β-OHB was analyzed with a commercially available ketone body meter (Keto-Mojo, Keto-Check, INC.) with blood obtained from the tail tip. After anesthesia and removal of the heart, approximately 1 mL of blood was harvested from the chest cavity and added to a microcentrifuge tube containing 0.5 mM EDTA. A small aliquot of whole blood was reserved for the hemoglobin A1c analysis, and the remaining blood was centrifuged at 2000× *g* for 20 min at 4 degrees Celsius. After centrifugation, the serum was separated and stored at −80 °C. Hemoglobin A1c was measured in whole blood using a commercially available kit (Crystal Chem Inc., Elk Grove Village, IL, USA). Fatty acids, triglycerides, and cholesterol were assessed in serum using a commercially available kit (Wako diagnostics). Insulin serum levels were assessed with a commercially available kit (Crystal Chem Inc., Elk Grove Village, IL, USA). Serum 17-beta estradiol was measured using a commercially available kit (#108667, Abcam, Waltham, MA, USA). All assays were performed according to the manufacturer’s instructions. Absorbance values from the colorimetric assays were read on a spectrophotometer (SmartReader96 ACCURIS Instruments, Edison, NJ, USA).

### 4.9. Tissue Analysis

The concentration of glycogen and triglycerides in the quadriceps muscle and liver was measured in frozen tissues harvested from mice, as indicated above, using colorimetric assays based on the manufacturer’s instructions. For glycogen measures, 20–30 mg of muscle and liver tissue was added to potassium hydroxide and incubated at 95 °C for 1 h. After the addition of sodium sulfate and 100% ethanol, the solution was incubated overnight at 4 °C. After centrifugation and rinsing with 80% ethanol, the glycogen pellet was dried at 40 °C overnight. Glucose released from glycogen was measured using amyloglucosidase and a glucose assay kit (GAHK-20, Sigma-Aldrich, St. Louis, MO, USA). For triglycerides, ~20–30 mg of muscle and liver were homogenized using stainless-steel beads in a surfactant solution provided by the manufacturer of the assay kit (#10010303, Cayman Chemicals, Ann Arbor, MI, USA). Concentrations were calculated using a standard curve and normalized to frozen tissue weights.

### 4.10. Gene Expression Analysis

RNA was extracted from the quadriceps tissue using the RNeasy Mini Kit (Qiagen #74104, Hilden, Germany). cDNA synthesis was performed using the Omniscript reverse transcriptase and random hexamers according to the manufacturer’s instructions (Omniscript RT kit, Qiagen). A real-time polymerase chain reaction (RT-PCR) analysis was performed using SYBR-Green. The mRNA levels of genes involved in glucose metabolism (glucose transporter 1 (*Glut1*), glucose transporter 4 (*Glut4*), insulin receptor substrate 1 (*Irs1*), insulin receptor substrate 2 (*Irs2*), and protein kinase B (*Akt1*)), ketone body metabolism (3-hydroxybutyrate dehydrogenase 1 (*Bdh1*), 3-oxoacid CoA-transferase 1 (*Oxct1*), and acetyl-CoA acetyltransferase 1 (*Acat1*)), and fatty acid metabolism (peroxisome proliferator-activated receptor alpha (*Pparα*), cluster of differentiation 36 (*Cd36*), medium-chain acyl-CoA dehydrogenase (*Mcad*), and diacylglycerol acyltransferase 1 (*Dgat1*)) were normalized to 18s rRNA. Primer sequences used for RT-PCR are presented in Table S1. Values were calculated as the fold change over the control-sedentary (CON-SED) group.

### 4.11. Statistical Analysis

All data are reported as mean ± standard error of the mean (SEM). Analyses involving two groups were conducted with Student’s *t*-test. Four group comparisons were analyzed using a two-way analysis of variance (ANOVA). Tukey’s range test was used for post hoc analysis. Statistical significance was tested at the *p* < 0.05 level. All graphs and analyses were performed using GraphPad Prism version 9.0 for Windows (GraphPad Software, San Diego, CA, USA).

## Data Availability

The authors declare that the data supporting the findings of the study are available within the article.

## Supplementary Materials

The following supporting information can be downloaded at: https://www.mdpi.com/article/10.3390/metabo12100948/s1, Table S1: Primer sequences for genes analyzed via RT-PCR.
