# Supplementary material for: The Effects of Exercise Training on Glucose Homeostasis and Muscle Metabolism in Type 1 Diabetic Female Mice
Source: Metabolites. 2022 Oct 5;12(10):948. doi: 10.3390/metabo12100948 (PMC9608674; doi:10.3390/metabo12100948)
Supplement: Supplementary file 1 [file metabolites-12-00948-s001.zip › metabolites-1683303-supplementary.pdf]

Table S1. Primer Sequences for Genes Analyzed via RT-PCR.

Gene	Gene ID	Primer	Sequence
Acat1	110446	Sense	GGCGCAGGTTTACCTATTT
		AntiSense	GTTGCTCCTCTGCTCATTAC
Akt1	11651	Sense	GCCTGATCAAGTTCTCCTACTC
		AntiSense	TGCCCACAGTAGAAACATCC
Bdh1	71911	Sense	GTGACCAACTCTGTACCTTCTC
		AntiSense	CATCTTCACCCAGACCTAACTC
Cd36	12491	Sense	ACTGGTGGATGGTTTCCTAGCCTT
		AntiSense	TTTCTCGCCAACTCCCAGGTACAA
Dgat1	13350	Sense	GGCCTTACTGGTTGAGTCTATC
		AntiSense	GTTGACATCCCGGTAGGAATAA
Glut1	20525	Sense	CATCGTGGCCATCTTTGGCTTTGT
		AntiSense	GGAAGCACATGCCCACAATGAAGT
Glut4	20528	Sense	TCGTGGCCATATTTGGCTTTGTGG
		AntiSense	AGGACCCATAGCATCCGCAACATA
Irs1	16367	Sense	TCCCTTTCTCCAGCACTAAC
		AntiSense	CCCTGTAGCCAAGAGGTATAAG
Irs2	384783	Sense	CTGGATAGAGGACTGAGGAAGA
		AntiSense	TCCTCTGGGTAAGGGTTGTA
Mcad	384783	Sense	TGGCGATGAAGGTTGAACTCGCTA
		AntiSense	GCTGATTGGCAATGTCTCCAGCAA
Ppara	19013	Sense	GCGTACGGCAATGGCTTTATCACA
		AntiSense	GCAGCCACAAACAGGGAAATGTCA
Oxct1	67041	Sense	AGCTTCCTTGCCTTGGTATC
		AntiSense	CATCCACTAAAGCAGTCCTACC
